# Prevalence and Risk Factors of Gastroesophageal Reflux Disease Among University Students in Saudi Arabia: A Systematic Review and Meta-Analysis of Studies Up to 2025

**DOI:** 10.7759/cureus.100121

**Published:** 2025-12-26

**Authors:** Osamah Hussain M Abu Hawi, Fatimah Hassan M Alqahtani, Fahad Khalid Almubarak, Alhanoof Ali Alyami, Nouf Safar S Al Shahrani, Nawal M Asiri, Raghad Jubran Alqahtani, Mohammed Shari Alshahrani

**Affiliations:** 1 Internal Medicine, Ahad Rufaidah General Hospital, Ahad Rafidah, SAU; 2 General Practice, King Faisal Specialist Hospital & Research Centre, Riyadh, SAU; 3 General Practice, King Saud Bin Abdulaziz University for Health Sciences, Riyadh, SAU; 4 General Practice, Khamis Mushayt General Hospital, Khamis Mushait, SAU; 5 General Practice, Armed Forces Hospital - Southern Region, Khamis Mushait, SAU; 6 College of Medicine, King Khalid University, Abha, SAU; 7 General Practice, Prince Sultan Military Medical City, Riyadh, SAU; 8 Diagnostic Radiology, Armed Forces Hospital - Southern Region, Khamis Mushait, SAU

**Keywords:** gastroesophageal reflux disease (gerd), prevalence of gerd, risk factors, saudi arabia, systematic review and meta analysis

## Abstract

Gastroesophageal reflux disease (GERD) is increasingly reported among young adults, yet its burden in university populations within Saudi Arabia has not been fully characterized. This systematic review and meta-analysis assessed the prevalence of GERD and its associated risk factors among university students in Saudi Arabia. A comprehensive search of PubMed, Scopus, Web of Science, and Google Scholar was conducted from inception to October 2025. Eligible studies were observational, conducted among university or college students in Saudi Arabia, and used validated diagnostic methods such as the GERD Questionnaire (GerdQ) with a cutoff score of ≥8. Eight studies involving 7,229 students met the inclusion criteria. Study characteristics, prevalence estimates, and associated risk factors were extracted, and study quality was assessed using the Newcastle-Ottawa Scale, with most studies demonstrating high methodological quality (scores 7-9). Meta-analysis was performed using random-effects models with logit transformation. The pooled prevalence of GERD was 26% (95%CI: 21-31%), with individual study estimates ranging from 18% to 35%. Subgroup analyses showed comparable prevalence estimates among female participants (24.97%), male participants (25.50%), medical students (29.30%), and non-medical or mixed student populations (25.43%), with no statistically significant differences between subgroups. Between-study heterogeneity was substantial (I² = 93.3%). Reported risk factors included smoking, high body mass index, tea or coffee consumption, fast food intake, psychological stress, and positive family history, while regular physical activity and higher fiber intake appeared protective. These findings indicate a substantial burden of GERD among university students in Saudi Arabia and highlight the need for targeted prevention and health promotion strategies in this population.

## Introduction and background

Gastroesophageal reflux disease (GERD) is a common chronic gastrointestinal disorder characterized by the reflux of gastric contents into the esophagus, leading to symptoms such as heartburn and regurgitation [1]. Although often associated with older adults, GERD affects individuals across all age groups, and its prevalence has been rising among young adults [2]. The condition can significantly impair quality of life by causing sleep disturbance, reduced productivity, and increased healthcare utilization, and may progress to complications such as erosive esophagitis, Barrett’s esophagus, or esophageal adenocarcinoma if not adequately managed [3].

As of 2017, global estimates indicate that GERD remains a substantial public health concern. While age-standardized prevalence changed little between 1990 and 2017, the absolute number of affected individuals increased markedly, largely due to population growth and aging [4]. A large meta-analysis reported a global pooled prevalence of GERD of approximately 14%, with substantial regional variation, ranging from 4% in China to over 22% in Turkey [5]. Such marked variation may reflect differences in diagnostic criteria, symptom definitions, study design, sampling strategies, genetic or ethnic susceptibility, and lifestyle patterns across populations. Higher prevalence estimates have been reported in several Middle Eastern and Eastern Mediterranean regions, particularly in Gulf Cooperation Council (GCC) countries and parts of the Levant, with reported prevalence ranging from 20% to 33%. These higher rates have been attributed to rising obesity levels, sedentary lifestyles, and increased consumption of fast food and caffeinated beverages [6].

Young adults in the region appear particularly affected. Studies among university students have documented high prevalence levels, including 32% in Oman and roughly 28% among Egyptian medical students [7,8]. Similar trends have been observed internationally, with prevalence exceeding 30% among university students in Ethiopia and Nigeria [9,10]. Across these populations, GERD has been consistently linked to modifiable behaviors common during university life, including smoking, elevated body mass index, frequent consumption of caffeine or soft drinks, irregular meal patterns, late-night eating, and psychological stress [8,10]. These factors reflect lifestyle changes and academic pressures characteristic of university environments, highlighting important opportunities for targeted preventive interventions.

In Saudi Arabia, the university student population has expanded rapidly, yet GERD within this group remains insufficiently characterized. Existing studies vary widely in diagnostic tools, sampling methods, and reported prevalence, making it difficult to determine the true burden of disease or identify consistent risk factors. A systematic review and meta-analysis is therefore needed to synthesize available evidence, provide reliable prevalence estimates, and clarify key behavioral and demographic determinants. Understanding GERD in this population is essential for informing targeted prevention and early intervention strategies, which may help mitigate long-term complications and reduce healthcare costs.

## Review

Methods

Study Design

This systematic review and meta-analysis aimed to determine the prevalence of GERD and its associated risk factors among university students in Saudi Arabia. The study was conducted in accordance with the Preferred Reporting Items for Systematic Reviews and Meta-Analyses (PRISMA) 2020 guidelines and the Cochrane Handbook for Systematic Reviews of Interventions [11,12]. The review protocol was not prospectively registered in the International Prospective Register of Systematic Reviews (PROSPERO), which is acknowledged as a methodological limitation.

The study was guided by an adapted PICO (Patient/Population, Intervention, Comparison, and Outcome) framework appropriate for prevalence and observational research [13]. The population of interest comprised university and college students enrolled in higher-education institutions in Saudi Arabia. The exposure was the presence of GERD, defined using validated diagnostic instruments such as the Gastroesophageal Reflux Disease Questionnaire (GerdQ) or standardized symptom-based criteria. Comparators included students without GERD as well as subgroups defined by demographic or lifestyle characteristics. The outcomes assessed were the prevalence of GERD, sex-specific prevalence, prevalence among medical versus non-medical students, and associated risk factors.

Search Strategy

A comprehensive search of electronic databases, including PubMed, Scopus, Web of Science, and Google Scholar, was conducted from inception to October 2025. The search strategy used both controlled vocabulary terms and free-text keywords relevant to GERD and university students in Saudi Arabia. Keywords included variants of the terms gastroesophageal reflux disease, GERD, reflux symptoms, heartburn, university students, and Saudi Arabia. Boolean operators were used to combine search terms to ensure both sensitivity and specificity. Only human studies published in English were considered. To identify additional eligible studies, the reference lists of included articles were manually reviewed. In Google Scholar, the first three pages of search results were screened since the platform prioritizes the most relevant records.

Selection Criteria

Studies were included if they met all of the following criteria. They must have been conducted among university or college students enrolled at higher education institutions in Saudi Arabia. They must have reported the prevalence of GERD or examined its associated factors. They must have used an observational design such as a cross-sectional, cohort, or case-control study. They must have clearly defined GERD using a validated diagnostic tool such as the GerdQ or standardized symptom criteria. Only studies conducted on human participants and published in English were considered eligible. Studies were excluded if they were conducted outside Saudi Arabia or examined populations that did not include university students. Additional exclusion criteria included review articles, case reports, editorials, conference abstracts lacking sufficient data, and studies without a clear definition or measurement of GERD. When duplicate publications were identified, the most comprehensive or recent version of the dataset was included.

Study Selection

All search results were imported into the Rayyan platform for reference management and duplicate removal [14]. Title and abstract screening were conducted independently by two reviewers. Full texts of potentially eligible studies were then assessed by four reviewers using the predefined inclusion and exclusion criteria. Any disagreements were resolved through group discussion or consultation with a senior reviewer. The screening and selection process is summarized in the PRISMA flow diagram.

Data Extraction

Data extraction was performed independently by four reviewers using a standardized Excel form (Microsoft Corporation, Redmond, Washington, United States). Extracted information included study characteristics such as author name, year of publication, city or region, university type, and study design. Additional variables included sample size, mean age or age range, sex distribution, academic major or college type, and the diagnostic method used to define GERD. Outcome measures included overall GERD prevalence, sex-specific prevalence, prevalence among medical and non-medical students, and reported risk factors such as smoking, BMI, dietary habits, caffeine consumption, stress, sleep patterns, and physical activity. When clarification was needed, attempts were made to contact corresponding authors. Extracted data were cross-checked by the review team to ensure accuracy and completeness.

Quality Assessment

The methodological quality of included studies was assessed using the Newcastle-Ottawa Scale (NOS) for observational studies [15], evaluating participant selection, comparability, and outcome ascertainment. Studies were categorized as high (7-9), moderate (4-6), or low quality (0-3). Four reviewers conducted assessments independently, with discrepancies resolved by consensus.

Statistical Analysis

All statistical analyses were conducted using R software version 4.3.3 with the meta package (R Foundation for Statistical Computing, Vienna, Austria, https://www.R-project.org/). Pooled prevalence estimates were calculated using random effects models based on a logit transformation. Between-study variance was estimated using the maximum likelihood method. The results were presented as pooled prevalence with corresponding 95% confidence intervals (CIs). Heterogeneity was quantified using the I^2^ statistic, Cochran’s Q test, and the between-study variance τ^2^ [16]. When at least three studies were available for a subgroup, prediction intervals were also generated to estimate the expected range of prevalence in similar future populations. Subgroup analyses were performed to compare GERD prevalence between males and females and between medical and non-medical student populations. A two-sided p-value less than 0.05 was considered statistically significant.

Results

Study Selection

**Figure 1:**
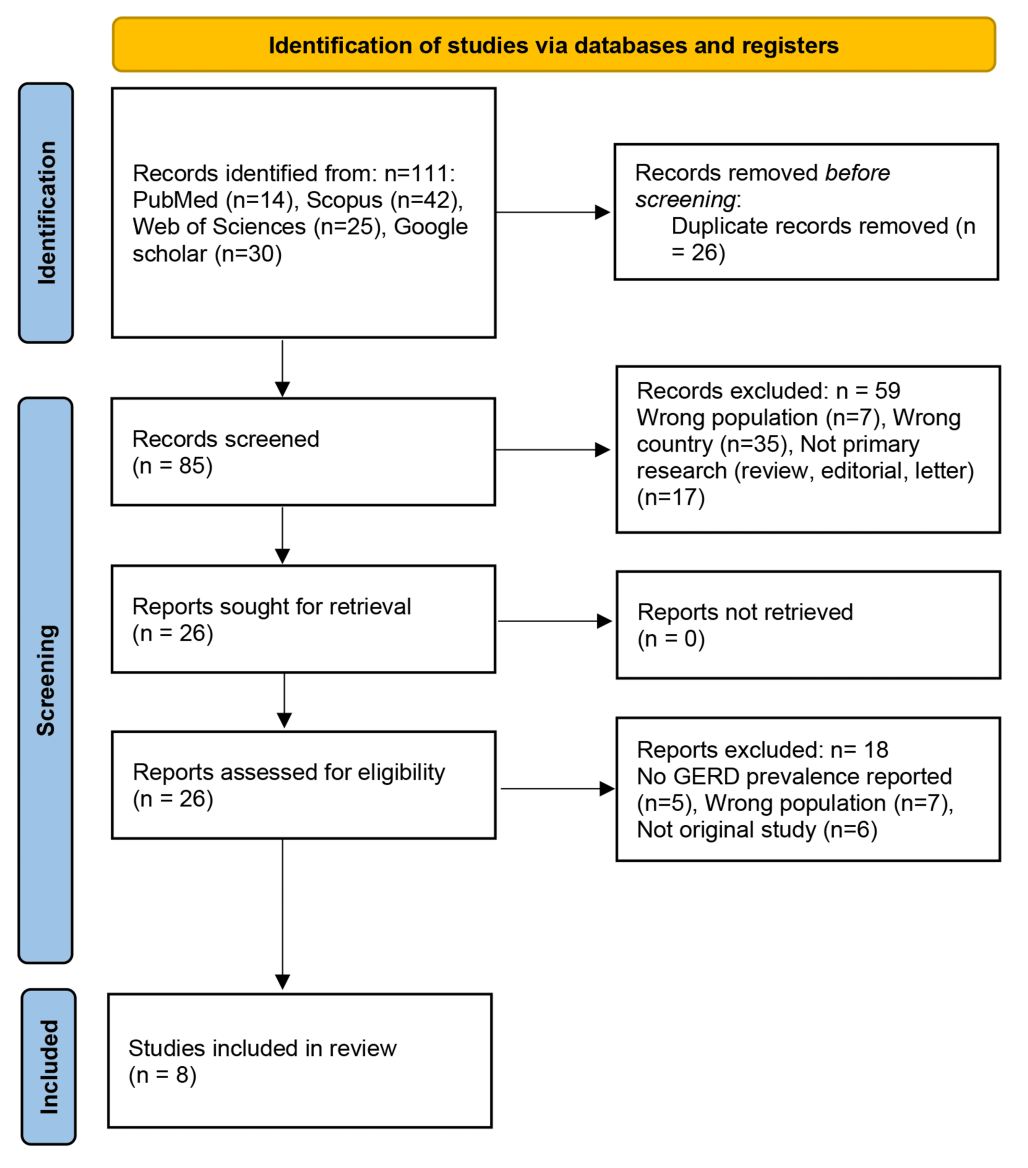
PRISMA flow diagram illustrating the selection process for studies included in the systematic review This flow diagram follows the PRISMA 2020 guidelines as described by Page et al. [12]. PRISMA: Preferred Reporting Items for Systematic Reviews and Meta-Analyses

A total of 111 records were identified through database searches, including PubMed (n = 14), Scopus (n = 42), Web of Science (n = 25), and Google Scholar (n = 30). After the removal of 26 duplicate records, 85 titles and abstracts were screened. Of these, 59 articles were excluded for the following reasons: wrong population (n = 7), wrong country (n = 35), or non-primary research such as reviews, editorials, or letters (n = 17). Full-text assessment was conducted for 26 articles, all of which were successfully retrieved. During this phase, 18 records were excluded due to the absence of GERD prevalence data (n = 5), ineligible population (n = 7), or being non-original studies (n = 6). Ultimately, eight studies met the eligibility criteria and were included in the final systematic review and meta-analysis (Figure 1).

Characteristics of Included Studies

Eight cross-sectional studies published between 2019 and 2024 were included, encompassing a total of 7,229 university students from various regions across Saudi Arabia (Table 1). Sample sizes ranged from 246 to 2,878 participants, and all studies employed validated symptom-based tools for GERD assessment, primarily the Gastroesophageal Reflux Disease Questionnaire (GerdQ) with a cutoff score of 8 or higher. The included populations represented diverse academic settings, including medical colleges, health and non-health colleges, and mixed university populations. GERD prevalence across individual studies varied widely, from 18.3% in Issa et al. [17] to 34.6% in Bin Abdulrahman et al. [18]. Several studies reported sex-specific findings: three studies demonstrated higher GERD prevalence among males, while one study reported a higher prevalence in females. Reported risk factors varied but consistently included lifestyle behaviors such as smoking, high BMI, fast food intake, tea or coffee consumption, stress, and positive family history. Some studies also identified protective factors such as regular physical activity and high fiber intake.

**Table 1:** Characteristics of included studies GERD was defined using the Gastroesophageal Reflux Disease Questionnaire (GerdQ) with a score ≥8 in all studies unless otherwise specified. PPI: proton pump inhibitor

Study (Year)	City or region	Population & sample size	Study design	Methods / tools used	GERD prevalence	GERD in Males vs Females	Notable findings
Awadalla, 2019 [19]	Southwestern Saudi Arabia (KKU)	2,878 students	Cross-sectional (stratified cluster sampling)	Questionnaire, GerdQ, Perceived Stress Scale	33.2%	Higher in males	Major factors: male sex, current/ex-smoking, non-health colleges, high stress.
Alrashed et al., 2019 [20]	Shaqra	400 students	Cross-sectional	Structured questionnaire + GerdQ	23.8%	M: 30.8% vs F: 14.5%	Risk factors: smoking, family history, BMI, fast food, tea, carbonated drinks, quick eating, sleeping soon after meals.
Al-Towairqi et al., 2020 [21]	Taif	256 female medical students	Cross-sectional	Online questionnaire + GerdQ	29.3%	Female-only study	Only significant factor: tea/coffee intake.
Issa et al., 2022 [17]	Multi-region (Riyadh, Taibah, Shaqra)	246 students	Cross-sectional (multistage sampling)	Online questionnaire + GerdQ	18.3%	No sex differences	Only significant factor: family history.
Otayf et al., 2022 [22]	Jazan	953 students	Cross-sectional	Arabic questionnaire + GerdQ	23.1%	Not reported	Risk factors: family history, >3 meals/day, PPI use; protective: physical activity, fiber-rich foods.
Alturki et al., 2023 [23]	Jeddah	397 university students	Cross-sectional	Online questionnaire + GerdQ	19.9%	F: 79.7% vs M: 20.3%	High e-cigarette use, but no smoking association; asthma linked to GERD.
Agwa et al., 2023 [24]	Al-Baha	566 students	Cross-sectional	Questionnaire on symptoms, risk factors, complications	25.44%	No sex-specific prevalence	Significant: smoking, alcohol, stress, spicy/fatty foods, coffee.
Bin Abdulrahman et al., 2024 [18]	Riyadh	1,533 undergraduate students	Cross-sectional	Online questionnaire + GerdQ	34.6%	M: 34.4% vs F: 34.6%	Higher prevalence in older ages, higher academic years, BMI, height association.

Overall Pooled Prevalence of GERD

**Figure 2:**
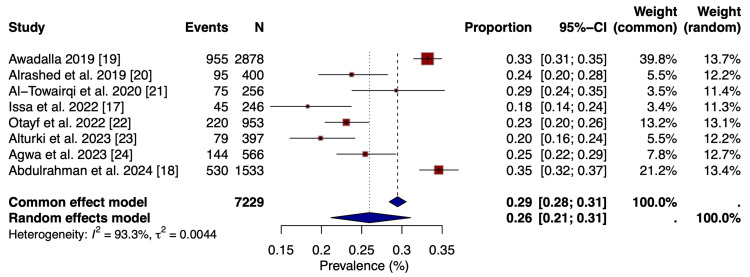
Forest plot showing the pooled prevalence of GERD References: [17-24] GERD: gastroesophageal reflux disease

The eight included studies provided data from 7,229 university students across different regions of Saudi Arabia. Individual study prevalence estimates ranged from 18% to 35% (Figure 2). Meta-analysis using a random-effects model produced a pooled GERD prevalence of 26% (95%CI: 21-31%), while the common-effects model yielded a similar estimate of 29% (95%CI: 28-31%). Considerable heterogeneity was observed among studies (I² = 93.3%, τ² = 0.0044). As I² values greater than 75% are generally considered indicative of substantial heterogeneity, the use of a random-effects model was appropriate to account for between-study variability in prevalence estimates across different study settings and populations. The forest plot illustrates the distribution of individual study estimates and the overall pooled effect.

Subgroup Analyses

Subgroup meta-analyses were conducted to explore variations in GERD prevalence (Table 2). Among female participants (four studies; n = 1,757), the pooled prevalence was 24.97% (95%CI: 14.61-39.30%), with substantial heterogeneity (I² = 91.6%). Male students (three studies; n = 829) demonstrated a comparable pooled prevalence of 25.50% (95%CI: 8.55-55.61%), with high heterogeneity (I² = 89.3%). Only one study evaluated medical students exclusively (n = 256), reporting a GERD prevalence of 29.30% (95% CI: 24.05-35.16%); therefore, robust conclusions regarding differences between medical and non-medical students could not be drawn. Non-medical or mixed university populations showed a pooled prevalence of 25.43% (95% CI: 20.44-31.15%) across seven studies, with substantial heterogeneity (I² = 93.9%).

**Table 2:** Subgroup meta-analysis of GERD prevalence among university students GERD: gastroesophageal reflux disease

Subgroup	k (studies)	Total participants	Events (cases)	Pooled prevalence (%)	95% CI (%)	Prediction interval (%)	I² (%)	τ²
Females	4	1,757	527	24.97	14.61–39.30	7.52–57.65	91.6	0.1522
Males	3	829	251	25.50	8.55–55.61	2.81–80.19	89.3	0.2386
Medical students	1	256	75	29.30	24.05–35.16	—	—	—
Non-medical or mixed	7	6,973	2,069	25.43	20.44–31.15	13.78–42.11	93.9	0.0826

Quality assessment

Quality appraisal using the NOS indicated that seven studies achieved high methodological quality (scores 7-9) and one study demonstrated moderate quality (score 6) (Table 3). Most studies received full scores in the selection domain due to adequate sampling strategies and clearly defined study populations. Comparability scores were generally lower because adjustment for important potential confounders, such as age, BMI, smoking status, dietary habits, physical activity, and socioeconomic characteristics, was limited or inconsistently reported across studies. Outcome and exposure assessment were strong across all studies, as all used validated GERD diagnostic tools. Overall, the quality of evidence was considered acceptable, with minimal risk of bias affecting the primary outcomes.

**Table 3:** Quality assessment using the Newcastle–Ottawa Scale (NOS) Quality assessment was conducted using the NOS adapted for observational cross-sectional studies, evaluating three domains: selection (maximum 4 points), comparability (maximum 2 points), and outcome/exposure assessment (maximum 3 points). Studies were classified as high quality (7–9 points), moderate quality (4–6 points), or low quality (0–3 points). Scores reflect methodological rigor based on sampling methods, control of confounders, and validity of GERD assessment. The use and structure of the NOS are based on the methodological evaluation described by Stang [15]. GERD: gastroesophageal reflux disease

Study (Year)	Selection (Max 4)	Comparability (Max 2)	Outcome/Exposure (Max 3)	Total score (Max 9)	Quality rating
Awadalla, 2019 [19]	4	2	3	9	High
Alrashed et al., 2019 [20]	4	1	3	8	High
Al-Towairqi et al., 2020 [21]	3	1	3	7	High
Issa et al., 2022 [17]	3	1	3	7	High
Otayf et al., 2022 [22]	4	1	3	8	High
Alturki et al., 2023 [23]	3	1	3	7	High
Agwa et al., 2023 [24]	3	1	2	6	Moderate
Bin Abdulrahman et al., 2024 [18]	4	1	3	8	High

Discussion

The​‍​‌‍​‍‌​‍​‌‍​‍‌ results of this systematic review and meta-analysis reveal that GERD is a frequent occurrence among university students in Saudi Arabia. The eight studies included in the review reported prevalence rates ranging from 18% to 35% in different individuals, which gave rise to a pooled prevalence of 26%. Several studies pointed to lifestyle and behavioral factors as major causes of GERD mechanisms. These factors included smoking, high BMI, consumption of caffeinated or fatty foods, soft drinks, irregular meal patterns, and psychological stress. Moreover, some protective factors, like regular physical activity and higher fiber intake, were also mentioned. Subgroup analyses have shown that the prevalence of GERD was quite similar in different genders and academic disciplines, indicating that students are still facing a heavy and widespread burden all over the ​‍​‌‍​‍‌​‍​‌‍​‍‌country [25].

The​‍​‌‍​‍‌​‍​‌‍​‍‌ pooled prevalence of 26% among Saudi university students matches the results of several regional and international studies. University student populations in Egypt have shown GERD prevalence rates ranging from 17% to 28% [7]. The factors that influenced the rates were stress, family history, and irregular dietary habits [26]. Indian studies reveal that the prevalence ranges from 11% to 20% in medical, engineering, and non-medical students [27]. Also, carbonated beverages, caffeine intake, lack of sleep, smoking, and late evening meals are some of the factors significantly associated with the condition [28]. The prevalence of the disease in Ethiopia has gone to 32%, where female sex, soft drink consumption, urban residence, and analgesic use, among other factors, are identified as the main causes of the disease [10].

These figures reflect a pattern observed worldwide. A comprehensive global review conducted by Nirwan and colleagues explored 102 studies from 37 countries and came up with a pooled global prevalence of GERD of 13.98% [5]. There were significant differences between regions revealed by their investigation, for example, the lowest prevalence in China, with 4.16%, and more than 22% in Turkey. In addition, there is a considerable impact of the modifiable lifestyle factors, for instance, obesity, smoking, and dietary habits [29]. Notwithstanding the global prevalence, which is lower than that of Saudi university students, the 26% difference may account for behaviors specific to certain ages, increased stress levels, and lifestyle changes, which are typical for young adults in higher education [30]. Together, this evidence suggests that GERD in university populations is influenced by a combination of local dietary patterns, academic stress, and global lifestyle trends that amplify risk among young adults.

Several factors reported in the included studies appear to contribute to the elevated burden of GERD in students. These include the dietary habits, BMI, smoking, caffeine consumption, stress, and irregular sleep patterns. Such results agree with those reported in other countries such as Oman [8], Ethiopia [10], India [27,31], Nigeria [9], and Italy, where the prevalence of GERD in university students ranged from 11% to 33%. For instance, in Oman, GERD prevalence was 32%, and the main factors were fast food intake, skipping breakfast, soft drink consumption, and lying down after meals [8]. Similarly, studies from India [31] and Ethiopia [10] identified smoking, use of analgesics, inadequate sleep, soft drink intake, and stress as the major factors leading to the problem. These similarities indicate that GERD in university students is strongly linked to the behavioral and lifestyle changes that are easily overlooked in academic environments but are ​‍​‌‍​‍‌​‍​‌‍​‍‌modifiable [32].

The role of stress, in particular, appears to be significant across diverse cultural contexts. Many of the included Saudi studies found strong associations between GERD and psychological stress, academic pressure, or higher academic years [19-21]. This is consistent with findings from Egypt, where medical students experiencing high perceived stress were nearly four times more likely to report GERD symptoms [7]. Stress may influence gastrointestinal function through altered visceral sensitivity, increased gastric acid secretion, and stress-related behavioral changes, including unhealthy eating patterns, increased caffeine consumption, and irregular or delayed meal timing [33,34]. Given the rigorous academic demands placed on university students, stress reduction strategies, alongside interventions targeting associated lifestyle behaviors, may represent an important component of GERD prevention in this population [35].

Dietary habits also featured prominently in the risk profiles across studies. Frequent consumption of tea or coffee, spicy or fatty foods, carbonated beverages, and late-night meals were among the most commonly reported factors associated with GERD symptoms [35]. Similarly, international studies from Italy and the United Arab Emirates reported associations between GERD and consumption of processed foods, low fiber intake, and irregular meal timing [36]. Although effect sizes were not consistently reported across studies, the repeated identification of these dietary behaviors underscores their potential relevance as modifiable risk factors. These findings highlight the importance of incorporating dietary education and healthy eating initiatives into university-based health promotion programs.

Physical activity was also highlighted as an important protective factor. A recent meta-analysis from Saudi Arabia demonstrated that individuals with low physical activity levels have a significantly higher likelihood of developing GERD [37]. Findings from the studies included in this review support this observation, with regular exercise consistently associated with a lower risk of reflux symptoms. Given the sedentary behaviors common among university students, feasible interventions, such as campus wellness programs, subsidized gym memberships, structured walking or cycling campaigns, and integration of physical activity into student support services, may represent practical and effective strategies for reducing GERD burden in this population.

Given that many identified risk factors for GERD among university students are modifiable, particularly within medical school environments, institutional-level interventions may play a critical preventive role. In the Saudi medical education context, curriculum-embedded wellness modules, including education on sleep hygiene, stress management, and healthy dietary practices, may be particularly effective, as they can be integrated into existing professionalism or student support courses. On-campus mental health services, including confidential counseling and stress management programs, may help address psychological stress, which has been consistently associated with GERD symptoms [38]. Additionally, flexible scheduling strategies, such as limiting prolonged late-evening academic activities or optimizing clinical rotation hours where feasible, could mitigate sleep disruption and irregular meal patterns commonly reported by medical students. The use of digital health tools, such as mobile applications for sleep tracking, stress monitoring, or lifestyle education, may also represent a culturally acceptable and scalable approach within Saudi universities, given high smartphone utilization and increasing institutional support for digital health initiatives [39]. Importantly, many of these interventions are likely to be feasible within Saudi medical colleges, as they align with ongoing national efforts to enhance student well-being, mental health awareness, and preventive health strategies within higher education settings.

Limitations

This review has several limitations. Most included studies used cross-sectional designs, which preclude causal inference. Substantial heterogeneity was observed across studies, likely reflecting differences in sampling methods, university settings, and measurement tools. Several studies relied on self-reported questionnaires, which may introduce recall or reporting bias. Sex-specific and subgroup data were not consistently available, limiting the precision of pooled estimates. Although only English-language studies were included, this restriction may have had a limited impact given that English is the primary language of academic publication in Saudi Arabia. Potential publication bias cannot be excluded, as studies reporting null or lower prevalence estimates may have been less likely to be published. Finally, the number of eligible studies was relatively small, and some regions of Saudi Arabia were underrepresented, which may reduce the generalizability of the findings to all university populations.

## Conclusions

This systematic review and meta-analysis demonstrates that GERD is common among university students in Saudi Arabia, affecting approximately one in four students. Prevalence estimates were broadly comparable across sex and academic fields, although substantial between-study heterogeneity was observed. Multiple modifiable lifestyle-related factors, including smoking, elevated BMI, frequent consumption of caffeinated or fatty foods, and psychological stress, were consistently associated with GERD, while regular physical activity and higher fiber intake appeared to have a protective role. The considerable burden identified in this young population highlights the need for targeted, institution-based interventions that promote healthier dietary habits, stress management, and active lifestyles within university settings. Further multicenter, longitudinal studies are warranted to clarify causal relationships and to inform the development of effective, context-specific prevention strategies.
